# Genomic evidence for the first symbiotic Deferribacterota, a novel gut symbiont from the deep-sea hydrothermal vent shrimp *Rimicaris kairei*

**DOI:** 10.3389/fmicb.2023.1179935

**Published:** 2023-06-29

**Authors:** Li Qi, Mengke Shi, Fang-Chao Zhu, Chun-Ang Lian, Li-Sheng He

**Affiliations:** ^1^Institute of Deep-Sea Science and Engineering, Chinese Academy of Sciences, Sanya, China; ^2^University of Chinese Academy of Sciences, Beijing, China; ^3^Key Laboratory of Tropical Marine Ecosystem and Bioresource, Fourth Institute of Oceanography, Ministry of Natural Resources, Beihai, China

**Keywords:** Deferribacterota, gut microbiota, *Rimicaris kairei*, metagenomic, hydrothermal vent

## Abstract

The genus *Rimicaris* is the dominant organism living in hydrothermal vents. However, little research has been done on the functions of their intestinal flora. Here, we investigated the potential functions of Deferribacterota, which is dominant in the intestine of *Rimicaris kairei* from the Central Indian Ridge. In total, six metagenome-assembled genomes (MAGs) of Deferribacterota were obtained using the metagenomic approach. The six Deferribacterota MAGs (Def-MAGs) were clustered into a new branch in the phylogenetic tree. The six Def-MAGs were further classified into three species, including one new order and two new genera, based on the results of phylogenetic analysis, relative evolutionary divergence (RED), average nucleotide identity (ANI), average amino acid identity (AAI) and DNA–DNA hybridization (DDH) values. The results of the energy metabolism study showed that these bacteria can use a variety of carbon sources, such as glycogen, sucrose, salicin, arbutin, glucose, cellobiose, and maltose. These bacteria have a type II secretion system and effector proteins that can transport some intracellular toxins to the extracellular compartment and a type V CRISPR–Cas system that can defend against various invasions. In addition, cofactors such as biotin, riboflavin, flavin mononucleotide (FMN), and flavin adenine dinucleotide (FAD) synthesized by *R. kairei* gut Deferribacterota may also assist their host in surviving under extreme conditions. Taken together, the potential function of Deferribacterota in the hydrothermal *R. kairei* gut suggests its long-term coevolution with the host.

## 1. Introduction

The *Rimicaris* shrimp is dominant in many hydrothermal vents. There are at least ten species in this genus, including *R. chacei*, *R. paulexa*, *R. parva*, *R. susannae*, *R. exoculata*, *R. falkorae*, *R. hybisae*, *R. kairei*, *R. vandoverae*, and *R. variabilis*. They mainly live in the hydrothermal fields with some difference in distribution among these species. For examples, *R. exoculata* is mainly living at the hydrothermal vent of the Mid-Atlantic Ridge (MAR), *R. kairei* is mainly resident at the Central Indian Ridge (Watabe and Hashimoto, 2002), and *R. chacei* also lives in the Atlantic Ridge, but most at the periphery of *R. exoculata* aggregates (Apremont et al., 2018). Within the *Rimicaris* genus, *R. exoculata*, *R. kairei*, and *R. hybisae* have an enlarged branchial chamber with a highly dense population of epibiotic bacteria (Zbinden and Cambon-Bonavita, 2020). *R. exoculata* is reported to house a density of epibiotic bacteria in cephalothoracic chamber and modified mouthparts (Gebruk et al., 2000). Due to that, a body of studies has focused on the epibionts and their benefits to *Rimicaris* species. The main epibiotic communities in *R. exoculata* are *Gamma-*, *Alpha-*, *Beta-*, *Delta-*, *Zetaproteobacteria*, *Campylobacteria*, and *Bacteroidetes* (Zbinden et al., 2008; Petersen et al., 2010; Hügler et al., 2011; Guri et al., 2012; Jan et al., 2014; Jiang et al., 2020). A few studies have also reported the gut microbial composition of *R. exoculata*. Three major groups, *Deferribacteres*, *Mollicutes*, and *Campylobacteria*, were found in the midgut, along with small amounts of *Gammaproteobacteria* (Zbinden and Cambon-Bonavita, 2003; Durand et al., 2010; Cowart et al., 2017). Additionally, the three main lineages were still present after 72 h of starvation, so they have designated resident epibionts rather than transient microflora (Durand et al., 2010). Besides, Apremont et al. showed that *Deferribacteres*, *Mollicutes*, and *Epsilon-* and *Gammaproteobacteria* were the main microbes existed in the digestive tract of *R. chacei* (Apremont et al., 2018). The difference between the intestinal microbial composition of *R. exoculata* and *R. chacei* may be due to the different living environments. In our previous studies, we showed that the intestinal microbiota of *R. kairei*, which was from the Central Indian Ridge, was dominant by Deferribacterota, Campylobacter, Bacteroidetes, Firmicutes, and Proteobacteria, although there is a significant difference in the composition between different developing stages (Qi et al., 2021). To date, some studies have been reported on the intestinal microbial community of *Rimicaris* species, but there are few studies on their functions, even though the functions of the gill symbiotic microbiota have been extensively investigated. Therefore, we will further investigate the functions of the gut microbiota and present the relationship with their host *Rimicaris* species.

Deferribacteres, a new phylum recorded in 2001 (Garrity et al., 2001), was emended in 2009 to represent only the family *Deferribacteriaceae* (Jumas-Bilak et al., 2009). In 2011, the phylogeny of the phylum was repositioned (Kunisawa, 2011), and the new name Deferribacterota was given in 2018 (Whitman et al., 2018). At present, there are few reports on the genomic function of Deferribacterota. Members of the phylum Deferribacterota are organized into a single order and six families. A deep lineage is the *Deferribacteraceae* family, whose genus *Deferribacter* includes four species, *D. thermophilus*, *D. desulfuricans*, *D. abyssii*, and *D. autotrophicus*. *D. thermophilus* was isolated from a high-temperature around 60°C, seawater-flooded oil reservoir in the North Sea (Greene et al., 1997), while the other three species were all isolated from deep-sea hydrothermal vents (Miroshnichenko et al., 2003; Takaki et al., 2010; Slobodkin et al., 2019). *D. desulfuricans* was obtained from the Suiyo Seamount hydrothermal chimney (Takaki et al., 2010), *D. abyssi* was isolated from the Rainbow hydrothermal vent field of the Mid-Atlantic Ridge (Miroshnichenko et al., 2003), and *D. autotrophicus* was isolated from Ashadze hydrothermal chimney on the Mid-Atlantic Ridge at a depth of 4,100 m (Slobodkin et al., 2019). All recognized *Deferribacter* species are strictly anaerobic and thermophilic organisms, which can oxidize various complex organic compounds and organic acids in the presence of diverse electron acceptors. *D. desulfurican* can use formate, acetate, and pyruvate as substrates; *D. abyssii* is capable of using molecular hydrogen, acetate, succinate, pyruvate, and proteinaceous compounds as electron donors and elemental sulfur, nitrate, or Fe (III) as electron acceptors; *D.autotrophicu* used molecular hydrogen, acetate, lactate, succinate, pyruvate and complex proteinaceous compounds as electron donors, and Fe (III), Mn (IV), nitrate or elemental sulfur as electron acceptors; *D. thermophilus* obtained energy from the reduction of manganese (IV), iron(III), and nitrate in the presence of yeast extract, peptone, casamino acid, tryptone, hydrogen, malate, acetate, citrate, pyruvate, lactate, succinate, and valerate. *D. desulfurican* is a heterotrophic bacterium, while all of the other three species are chemolithoautotrophic bacteria. In addition, *Flexistipes sinusarabici*, which was most closely related to *Deferribacter* and isolated from the Atlantis Deep brines of the Red Sea, is tolerant to high temperature, high salt concentration and heavy metals, and strictly anaerobic. This organism prefers complex growth substrates such as yeast extract, meat extract, peptone, and trypsin, while formate, lactate, citrate, malate, carbohydrate, amino acid, and alcohol do not support cell growth (Lapidus et al., 2011). Up to now, only one species of *Mucispirillum schaedleri* in the phylum of Deferribacterota has been isolated from the intestine. *M. schaedleri* inhabits the intestinal mucus layer of rodents and other animals in abundance and are considered pathogenic. *M. schaedleri* harbors a complete Embden-Meyerhof-Parnas (EMP) pathway and a nonoxidative pentose phosphate pathway as well as a complete tricarboxylic acid cycle. *M. schaedleri* can also alter gene expression in mucosal tissues, suggesting an intimate interaction with the host (Loy et al., 2017).

Different from the reported Deferribacterota in the hydrothermal zone, the six Deferribacterota from *R. kairei* guts are heterotrophic according to the MAG analysis. Def-MAGs do not have genes related to carbon, nitrogen, and sulfur utilization. Instead, they have a complete glycolysis pathway and genes for transporting and degrading polysaccharides. Although slight differences among the six Def-MAGs, the main functions of carbohydrate metabolism, polysaccharide degradation, vitamin synthesis and so on were similar. This study further elucidated the diversity of Deferribacterota and their host interaction relationships, supplementing the understanding of existing Deferribacterota.

## 2. Materials and methods

### 2.1. Sample collection

The shrimp were collected from two sites in the Central Indian Ridge by the manned submersible *Deep-Sea* Yongshi during R/V *Tansuoyihao* research cruise TS10 (February 2019). Sampling sites were located in Edmond (69.59667°E, 23.87782°S) and Kairei (70.04010°E, 25.32048°S) at depths of 3,281 m and 2,421 m, respectively (Supplementary Figure S1). All shrimp were obtained using the suction sampler. Once onboard, individuals were immediately frozen at −80°C or stored in 75% ethanol at −20°C.

### 2.2. DNA extraction and metagenome sequencing

The samples were identified as *R. kairei* in our previous article (Qi et al., 2021). The intestinal anatomy of *R. kairei* was performed in a sterile environment, and the total DNA of the gut was extracted using a PowerSoil DNA isolation kit (Qiagen, Germany) following the manufacturer’s procedures. The quality and quantity of genomic DNA were checked by gel electrophoresis. The DNA concentration was determined by using a Qubit dsDNA HS assay kit with a Qubit 2.0 fluorometer (Invitrogen, Carlsbad, CA). A total of 100 ng DNA was used for library preparation. High-throughput sequencing was performed with the Novaseq 6000 platform to produce 2 × 150 bp paired-end reads (Illumina).

### 2.3. Metagenomic assembly and genome binning

Metagenomic DNA sequencing produced a total of 154,387,934 reads, with a total length of 45 Gbp. Trimmomatic v0.36 was used for trimming with parameters (LEADING: 3; The TRAILING: 3; HEADCROP: 4; SLIDINGWIDOW: 4:15; MINLEN: 80) (Bolger et al., 2014), and FastQC v0.11.9 was used to evaluate the quality of data before and after filtering (Brown et al., 2017). The qualified reads were further assembled into contigs by SPAdes-3.11 (Bankevich et al., 2012) software with a k-mer range of 21 to 127. MetaWRAP v1.2.1 (Uritskiy et al., 2018) was used for genome binning and subsequent refinement with parameters (three different algorithms MaxBin2, metaBAT2, and CONCOCT for metagenomic binning; contig length: >2000 bp; completeness: >50%; and contamination: <10%). The MAGs (metagenome assembled genomes) were checked by CheckM v1.1.3 (Parks et al., 2015) to filter those with low completeness (<50%) and high contamination (>10%). Taxonomic annotation of the MAGs was performed using GTDB-tk v1.4.0 software (Chaumeil et al., 2019). The whole-genome average nucleotide identity (ANI) between genomes was calculated by fastANI v1.33 software (Jain et al., 2018). Average Amino acid Identity (AAI) was calculated by AAI calculator online tool (Rodríguez-R and Konstantinidis, 2014). DNA–DNA hybridization (DDH) was calculated by Genome-to-Genome Distance Calculator 3.0 online tool (Meier-Kolthoff et al., 2021). GTDB-tk software was used to calculate the relative evolutionary divergence (RED) values when a query genome could not be classified based on the ANI values. Then, the MAGs belonging to Deferribacterota bacteria were retrieved for downstream analyses.

### 2.4. Phylogenetic analyses

There were 79 genomes in total for phylogenetic analyses, including 57 genomes from Tenericutes, Firmicutes, Acidobacteria, Chrysiogenetes, and Proteobacteria, and the others were from the phylum Deferribacterota. Firstly, 43 ribosome proteins were obtained by CheckM analysis from all of these genomes and then aligned by MAFFT v7.487 (Katoh et al., 2002) with the default settings, and poorly aligned regions were removed by trimAI v1.4 (Capella-Gutiérrez et al., 2009). The maximum likelihood (ML) phylogenomic tree was constructed using the concatenated aligned protein sequences with the IQ-TREE tool (Nguyen et al., 2015) and the best-fit substitution model (LG + R10 model) for 1,000 replicates. In addition, all phylogenetic trees were visualized using the interactive Tree of Life (iTOL) online tool (Letunic and Bork, 2019).

### 2.5. Genome annotation

Reference genomes included *D. autotrophicus*, *D. desulfuricans* SSM1, *F. sinusarabici* DSM4947, and *M. schaedleri* ASF457, of which *D. autotrophicus*, *D. desulfuricans* SSM1 and *F. sinusarabici* DSM4947 were free-living bacteria from hydrothermal environments, and *M. schaedleri* ASF457 was from the mouse intestine. The studied MAGs and reference genomes were annotated using Prokka v1.14.6, and the parameters were set to metagenome and kingdom bacteria (Seemann, 2014). This annotation pipeline relied on several external prediction tools, including Prodigal for coding sequences, Aragon for transfer RNA genes, RNAmmer for ribosomal RNA genes, and Infernal for noncoding RNAs. Kofamscan v1.3.0 (Aramaki et al., 2020) was used for functional annotations of the predicted genes, and the amino acid sequences of each genome were inputted and then compared with the KEGG database using the mapper model. KEGG-Decoder was performed to determine the completeness of various metabolic pathways by Kofamscan results (Graham et al., 2018). GO annotation was analysed using eggNOG-mapper v2.1.3 software (Huerta-Cepas et al., 2017), and the parameters were set as follows: -seed_ortholog_evalue, 0.00001; −m, hmmer; and -d, bact. Carbohydrate enzyme annotation was performed by searching the CAZyme database using dbCAN2 software, which used default settings (Zhang et al., 2018). Annotation of the deduced proteins was also performed using BLASTP against the NCBI Nr, KEGG (Kanehisa et al., 2016), Pfam (El-Gebali et al., 2019) and COG (Tatusov et al., 2000; Galperin et al., 2021) databases, with a maximum e-value cut-off of 1e-05. The CRISPR region was identified using CRISPRCasTyper v1.6.1 (Russel et al., 2020) and the CRISPRCasFinder online tool (Grissa et al., 2007).

#### Data availability

The MAGs obtained from the samples of Gut of *Rimicaris kairei* in this study have been submitted to the NCBI database under BioProject ID PRJNA931729.

## 3. Results

### 3.1. Metagenome assembly and characteristics

Deferribacterota was the dominant phylum in both microbial communities of the juvenile and adult *R. kairei* intestinal tracts in our previous study (Qi et al., 2021). To further investigate the potential functions of the intestinal Deferribacterota, metagenomes were assembled and analysed. A total of 45 Gbps of raw data were obtained by high-throughput sequencing, and 42.4 Gbps of clean data were retained. After *de novo* assembly based on three different algorithms (MaxBin2, metaBAT2, and CONCOCT), genomic bins with each longer than 2 kbp, completeness >50%, and contamination <10% were selected. There were 18 MAGs obtained in total, including Firmicutes, Bacteroidetes, Spirochaetota, Campylobacterota and Deferribacterota, based on the analysis by GTDB-tk (Supplementary Table S1). The six Def-MAGs (Deferribacterota MAGs, defined as *Def_J1, Def_J3, Def_J5, Def_J6, Def_A4*, and *Def_A7*, respectively) with four related reference genomes, including *D. autotrophicus*, *D. desulfuricans* SSM1, *F. sinusarabici* DSM4947, and *M. schaedleri* ASF457, are shown in Table 1. Among the six Def-MAGs, the completeness of *Def_J6* is close to 90%, and its genome size is 2.5 Mb, similar to the four reference MAGs with a genome size of 2.2–2.5 Mb. The completeness of other Def-MAGs ranged from 55.33 to 88.76%, with genome sizes ranging from 0.9 to 1.8 Mb. Notably, the average G + C content of the six Def-MAGs ranged from 46.4 to 50.7%, much higher than that of the reference genomes, in which the G + C content ranged from 30 to 38%. We also listed the number of tRNAs and rRNAs and the number and percentage of genes annotated by each genome in the KEGG and COG databases (Table 1).

**Table 1:** Genomic features of gut Deferribacterota in *R. kairei* and reference genomes.

Genome features	*Def_J1*	*Def_J3*	*Def_J5*	*Def_J6*	*Def_A4*	*Def_A7*	*Mucispirillum schaedleri ASF457*	*Deferribacter autotrophicus*	*Deferribacter desulfuricans SSM1*	*Flexistipes sinusarabici DSM4947*
Habitat	*R. kairei* intestine	*R. kairei* intestine	*R. kairei* intestine	*R. kairei* intestine	*R. kairei* intestine	*R. kairei* intestine	Mouse caecal mucus	Hydrothermal vent	Hydrothermal vent	Brine water (Atlantis II Deep, Red Sea)
Genome size (bp)	1,364,439	1,821,084	927,196	2,565,562	1,567,595	1,248,453	2,332,248	2,542,980	2,234,389	2,526,590
G + C content (%)	49.6	50.2	49	46.4	49.3	50.7	31	32.6	30.3	38.3
CheckM Compeleteness (%)	78.19	88.76	55.33	90.05	75.13	71.92	98.28	96.55	96.55	99.14
CheckM Contamination (%)	0	0.143	0	0	0	1.754	2.59	2.59	1.72	0.86
Coding density (%)	83.4	82.2	86.6	71.6	83.5	81.9	87.3	90.8	93	88.4
Contig number (≥50,000 bp)	6	8	0	10	2	7	22	10	2	1
N50 (scaffolds)	39,456	43,507	5,119	22,155	10,807	38,451	530,101	319,692	2,234,389	2,526,590
CDS number	1,119	1,382	824	1743	1,309	935	2,162	2,488	2,470	2,356
tRNA genes	32	17	29	39	11	31	41	43	44	44
No.(%) of KEGG genes	685 (61.22)	896 (64.833)	413 (50.12)	725 (41.59)	710 (54.24)	539 (57.65)	1,319 (61.01)	1,521 (61.13)	1,540 (62.35)	1,518 (64.43)
No.(%) of COG genes	827 (73.91)	1,091 (78.94)	503 (61.04)	939 (53.87)	872 (66.62)	664 (71.02)	1719 (79.51)	2,121 (85.25)	2070 (83.81)	2,136 (90.66)

### 3.2. Phylogenetic analysis of Deferribacterota

**Figure 1:**
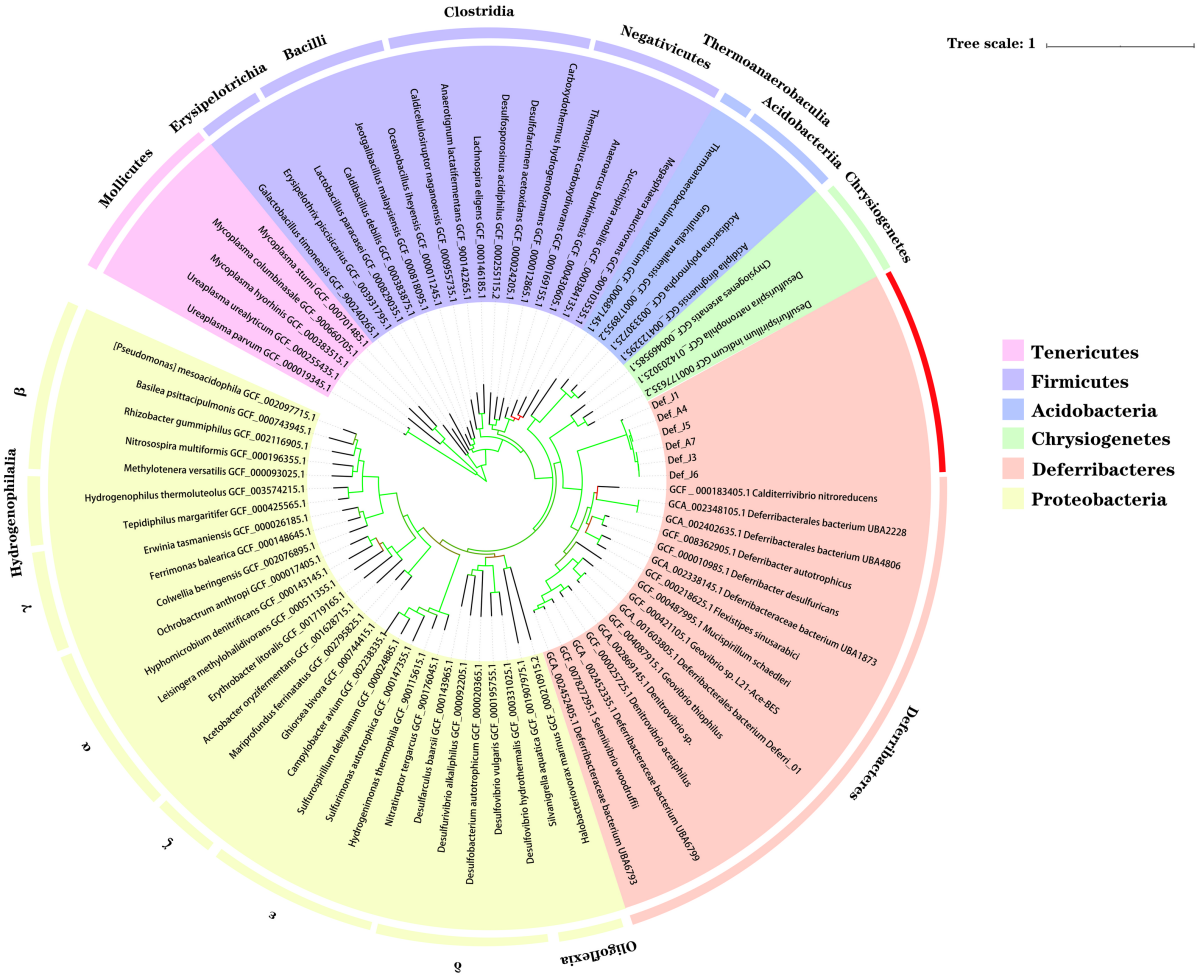
Phylogenetic position of gut Deferribacterota in *R. kairei*. Phylogenomic analysis was conducted on 43 concatenated conserved proteins to reconstruct a maximum-likelihood tree with 1,000 replications. Bootstrap values are colour-coded in the branches, with a colour range of red to green indicating values from 47 to 100. Different background colours represent different phyla. The ones marked in the outer circle are the class names, and the red ones are the samples of this study.

To further investigate the taxonomic status of the six species from the *R. kairei* intestine, phylogenetic analysis was performed using 43 conserved proteins with five phyla adjacent to the phylum Deferribacterota: Tenericutes, Firmicutes, Acidobacteria, Chrysiogenetes, and Proteobacteria. In addition to the six studied species from Deferribacterota, 16 other genome sequences in the phylum Deferribacterota, including 8 identified and 8 unidentified species, were selected from the RefSeq database, and a total of 79 genomes were constructed for phylogenetic analyses (Figure 1). The results showed that the six Def-MAGs were clustered into a single branch and separated from other strains in the phylum Deferribacterota. We also tested the average nucleotide identity (ANI) value (Table 2). The results, consistent with the phylogenetic tree, were that the six Def-MAGs clustered together (Supplementary Figure S2). The Average Amino acid Identity (AAI) and DNA–DNA hybridization (DDH) values are similar to the ANI values, and the six Def-MAGs are classified into three species (Table 2). According to the classification analysis by GTDB-tk software, *Def_J3*, *Def_J5*, *Def_J6*, and *Def_A7* are classified as the *Deferribacteres* class but could not be further classified into any known order. The relative evolutionary divergence (RED) values are 0.467, 0.460, 0.467, and 0.455 for *Def_J3*, *Def_J5*, *Def_J6*, and *Def_A7*, respectively. *Def_J1* and *Def_A4* are classified as the *Mucispirillaceae* family but could not be further classified into any known genus. The RED values were 0.747 and 0.749 for *Def_J1* and *Def_A4*, respectively (Table 2). Therefore, *Def_J3*, *Def_J5*, *Def_J6*, and *Def_A7* could be in new orders, while *Def_J1* and *Def_A4* could be in new genera. Together with the phylogenetic results and ANI values, the six Def-MAGs were classified into three species, with *Def_J1* representing a new genus, *Def_J3*, *Def_J6*, and *Def_A7* representing a new order, and *Def_J5* and *Def_A4* representing another new genus. Here, the classification of *Def_J5* is slightly confused, which is probably due to its genome with only 50% integrity and needs to be further clarified by obtaining a longer length.

**Table 2:** Average nucleotide identity, average amino acid identity, DNA–DNA hybridization and relative evolutionary branching values of gut Deferribacterota in *R. kairei*.

ANI	*Def_J1*	*Def_J3*	*Def_J5*	*Def_J6*	*Def_A4*	*Def_A7*
*Def_J1*	100%	81.22%	81.73%	81.16%	83.62%	80.99%
*Def_J3*	80.91%	100%	79.80%	98.79%	80.92%	98.33%
*Def_J5*	81.96%	79.72%	100%	80.51%	98.26%	79.54%
*Def_J6*	81.01%	98.91%	80.49%	100%	80.48%	98.53%
*Def_A4*	83.54%	80.74%	97.65%	80.70%	100%	80.03%
*Def_A7*	81.11%	98.66%	79.62%	98.58%	80.25%	100%
**AAI**	***Def_J1***	***Def_J3***	***Def_J5***	***Def_J6***	***Def_A4***	***Def_A7***
*Def_J1*	100%	78.37%	77.33%	78.48%	81.26%	78.23%
*Def_J3*	78.37%	100%	74.49%	97.69%	78.22%	96.92%
*Def_J5*	77.33%	74.49%	100%	73.49%	95.38%	73.61%
*Def_J6*	78.48%	97.69%	73.49%	100%	76.93%	96.60%
*Def_A4*	81.26%	78.22%	95.38%	76.93%	100%	75.91%
*Def_A7*	78.23%	96.92%	73.61%	96.60%	75.91%	100%
**DDH**	***Def_J1***	***Def_J3***	***Def_J5***	***Def_J6***	***Def_A4***	***Def_A7***
*Def_J1*	100%	23.60%	23.50%	23.10%	25.90%	24.40%
*Def_J3*	23.60%	100%	21.90%	76.30%	25.80%	69.70%
*Def_J5*	23.50%	21.90%	100%	23.30%	74.20%	22.00%
*Def_J6*	23.10%	76.30%	23.30%	100%	24.30%	70.90%
*Def_A4*	25.90%	25.80%	74.20%	24.30%	100%	22.80%
*Def_A7*	24.40%	69.70%	22.00%	70.90%	22.80%	100%
RED	0.7469	0.4667	0.4596	0.467	0.749	0.455
(GTDB)	Family	Class	Class	Class	Family	Class

ANI and AAI >95%, and DDH >70% are considered one species, both of them are marked in red.

### 3.3. Metabolic features

#### 3.3.1. Energy metabolism

**Figure 2:**
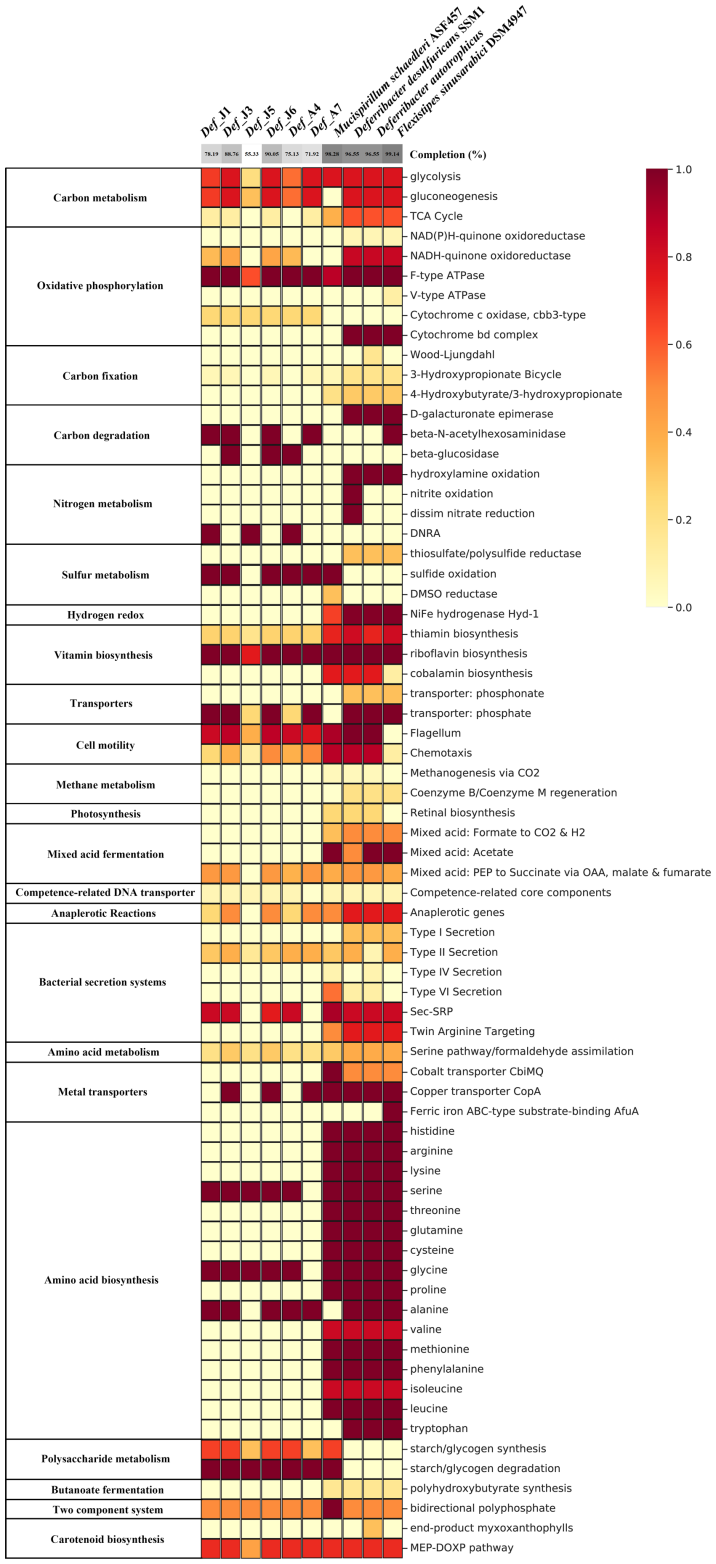
Heatmap based on KEGG annotation. The heatmap represents the integrity of the metabolic pathways of MAGs based on the presence or absence of genes identified by the KEGG decoder. The absence or presence of genes identified by the KEGG decoder. The grey colour at the top indicates the degree of genome integrity.

**Figure 3:**
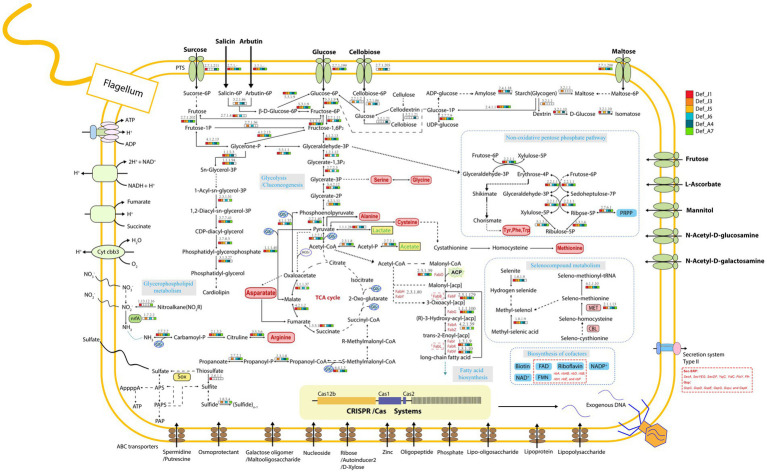
Diagram of the major predicted metabolic pathways. Metabolites are shown in black, amino acids in red, cofactors in blue, and the enzymes predicted for each MAG in each process are shown in different small blocks of colour in the diagram, with white indicating that the enzyme is not present. PTS, ABC transporters, CRISPR–Cas systems, etc., are indicated in the diagram.

Six Def-MAGs and four reference MAGs were used to comparatively analyse the metabolic pathways by KEGG decoding. Based on the heatmap, both groups have a similar integrity of metabolism for the glycolysis and gluconeogenesis pathways, but the gluconeogenesis pathway was almost absent in the genome of *M. schaedleri* ASF457 (Figure 2). Genes related to the tricarboxylic acid cycle were severely absent in Def-MAG compared to the reference MAGs (Figure 2). Taking *Def_J6*-MAG, with the highest completeness, as an example, only malate dehydrogenase (EC1.1.1.37), fumarate hydratase (EC4.2.1.2), and succinate dehydrogenase (ubiquinone) flavoprotein subunit (EC1.3.5.1) were found, and most of the enzymes, including key enzymes such as citrate synthase (EC2.3.3.1) and ATP citrate lyase (EC2.3.3.8), were absent. In Def-MAG and *M. schaedleri* ASF457, starch or glycogen has 33–66% integrity for the synthesis pathway and 100% integrity for the degradation pathway. However, both pathways were absent in the three environmental reference genomes (Supplementary Table S2). We found that all six Def-MAGs have the key enzyme glycogen phosphorylase (EC2.4.1.1.1), which is involved in glycogen degradation, while 1,4-α-glucan branching enzyme (EC2.4.1.18), which is essential for increasing the solubility of glycogen molecules and reducing the osmotic pressure within cells, is only present in *Def_J1*, *Def_J3*, *Def_J6* and *Def_A4* (Figure 3). Notably, the carbon degradation of the six Def-MAGs was mainly *via* β-N-acetylhexosaminidase and β-glucosidase, while the three environmental reference genomes were mainly *via* D-galacturonate epimerase, and the carbon degradation process was not present in the *M. schaedleri* ASF457 MAG from the mouse intestine (Figure 2). β-N-acetylhexosaminidase and β-glucosidase are key enzymes for the degradation of chitin and cellulose, respectively, while D-galacturonate epimerase is able to convert UDP-d-glucuronide into D-galacturonide, a monosaccharide that is one of the activating precursors necessary for the synthesis of pectinas, indicating that the six Def-MAGs from the *R. kairei* intestine are able to degrade cellulose and chitin, while the reference genomes have the potential to degrade pectin. Def-MAGs contain many phosphotransferases, which transport extracellular glucose (EC22.7.1.199), salicin (EC2.7.1.-), arbutin (EC2.7.1--), cellobiose (EC2.7.1.205), maltose (EC2.7.12.08), and other carbon sources into cells (Figure 3). The large number of PTSs identified in the Def-MAG genomes suggests that Def-MAG may utilize multiple carbon sources. In addition, we found that Def-MAG has carbamate kinase (EC2.7.2.2) and ornithine carbamoyltransferase (EC2.1.3.3) (Figure 3), both of which convert NH_3_ to citrulline and over the process reversibly. Although no nitric oxide synthase (EC1.14.14.17) was found for the conversion from citrulline to arginine, arginine deiminase (EC3.5.3.6), which catabolizes arginine to citrulline, is present and can irreversibly hydrolyse L-arginine to L-citrulline and ammonia, suggesting that Def-MAGs could provide energy by arginine metabolism generating ATP.

#### 3.3.2. Carbohydrate enzymes

**Figure 4:**
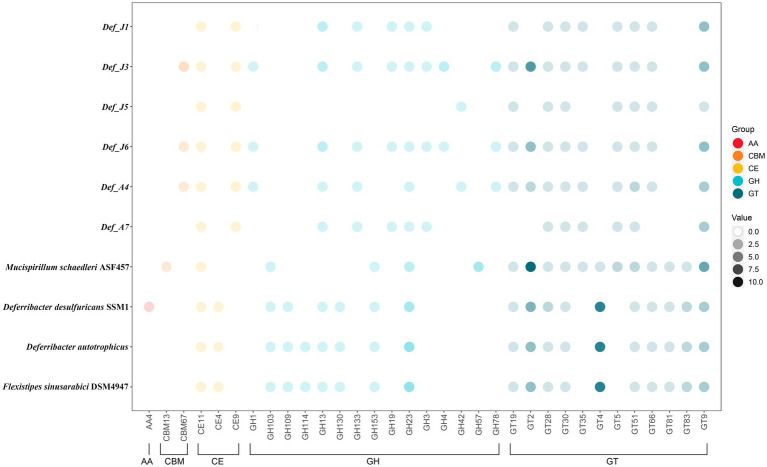
Relative abundance of carbohydrate-active enzyme genes. The different coloured circles represent the different types of enzymes, auxiliary activity (AA), carbohydrate-binding module (CBM), carbohydrate esterase (CE), glycoside hydrolase (GH), and glycosyltransferase (GT). The darkness of the circles indicates the number of each carbohydrate-active enzyme gene in different MAGs. Gene distribution, classification, and functions are reported in Supplementary Table S3.

In the six Def-MAGs and four reference genomes, a total of 33 carbohydrate-active enzymes were identified, which were classified into 5 types: AA (auxiliary activities), CBM (carbohydrate-binding modules), CE (carbohydrate esterases), GH (glycoside hydrolases) and GT (glycosyltransferases). Among them, a total of 21 carbohydrate enzymes were in six De-MAGs, nine of which were specific in the six Def-MAGs, including one CBM, CBM67; one CE, CE9; and 7 GHs, GH1, GH133, GH19, GH3, GH4, GH42, and GH78 (Figure 4). A total of 18 carbohydrate enzymes were identified in the genome of *M. schaedleri*, among which CBM13 and GH57 were specific (Figure 4). A total of 20 carbohydrate enzymes were identified in the genomes of the three environmental Deferribacterota isolates, five of which were specific to these genomes, namely, AA4, CE4, GH109, GH114, and GH130 (Figure 4). In addition, the six Def-MAGs were predominantly rich in glycoside hydrolases compared to the mouse intestine, where the main type of carbohydrase was glycosyltransferase. Compared with the environmental Deferribacterota genomes, the six Def-MAGs have specific GT35 and GT5 glycosyltransferases (Figure 4). These two glycosyltransferases can phosphorylate or synthesize glycogen, suggesting that Deferribacterota in the gut may provide a carbon source for the host.

#### 3.3.3. Amino acids, vitamins, and cofactors

Compared to the three environmental reference genomes, the six Def-MAGs lost most amino acid synthesis genes except serine, glycine, and alanine; however, for the genome of *M. schaedleri* ASF457, the *Deferribacterota* from the mouse intestine, only the alanine and tryptophan synthesis pathways were absent (Figure 2). This result suggests the interdependent relationship between Def-MAGs and their host *R. kairei*. Except for *Def_J5*, the integrity of the pathways involved in riboflavin synthesis was 100% in the remaining five Def-MAGs and four reference genomes (Supplementary Table S2), and the flavin-like compound biosynthesis-related genes *ribA*, *ribAB*, *ribD*, *ribB*, *ribH*, *ribE*, and *ribF* were identified in the six Def-MAG genomic metabolic pathway reconstructions (Figure 3). In addition, Def-MAGs also have biotin, flavin mononucleotide (FMN), flavin adenine dinucleotide (FAD) and other cofactor synthesis pathways (Figure 3).

#### 3.3.4. Secretome and immune protection

The secretory system is essential for prokaryotic cell growth and other physiological processes (Green and Mecsas, 2016). Compared with the reference genomes, the six Def-MAGs contained only a type II secretion system (T2SS), but the reference genomes also have type I, type IV, and type VI secretion systems (Figure 2). T2SS effectors are transferred from the cytoplasm to the outer membrane or into the extracellular environment in two steps. First, Protein translocation across the inner membrane *via* the general secretion (Sec) pathway or twin arginine translocation (Tat) pathway (Pugsley et al., 1991; Voulhoux et al., 2001). Second, protein folded in the periplasm crosses the outer membrane by transport of T2SS (Johnson et al., 2006; Douzi et al., 2011; Nivaskumar and Francetic, 2014). Key proteins of the Sec pathway and the signal recognition particle (SRP) pathway were blasted and found in six Def-MAGs, including SecA, SecYEG, SecDF, YajC, YidC, FtsY, and Ffh, but the Secret monitor (SecM) protein was absent. In Def-MAGs, six of different genral secretory pathway (Gsp) protein of T2SS were also found, namely, GspC, GspD, GspE, GspG, GspJ, and GspK (Figure 3). These results indicate that the protein secretion of Deferribacterota in the *R. kairei* intestine mainly transfers the protein to the periplasmic space through the Sec pathway, and then the protein folds, while the Tat pathway existing in reference genomes transfers the folded protein to the periplasmic space.

**Figure 5:**
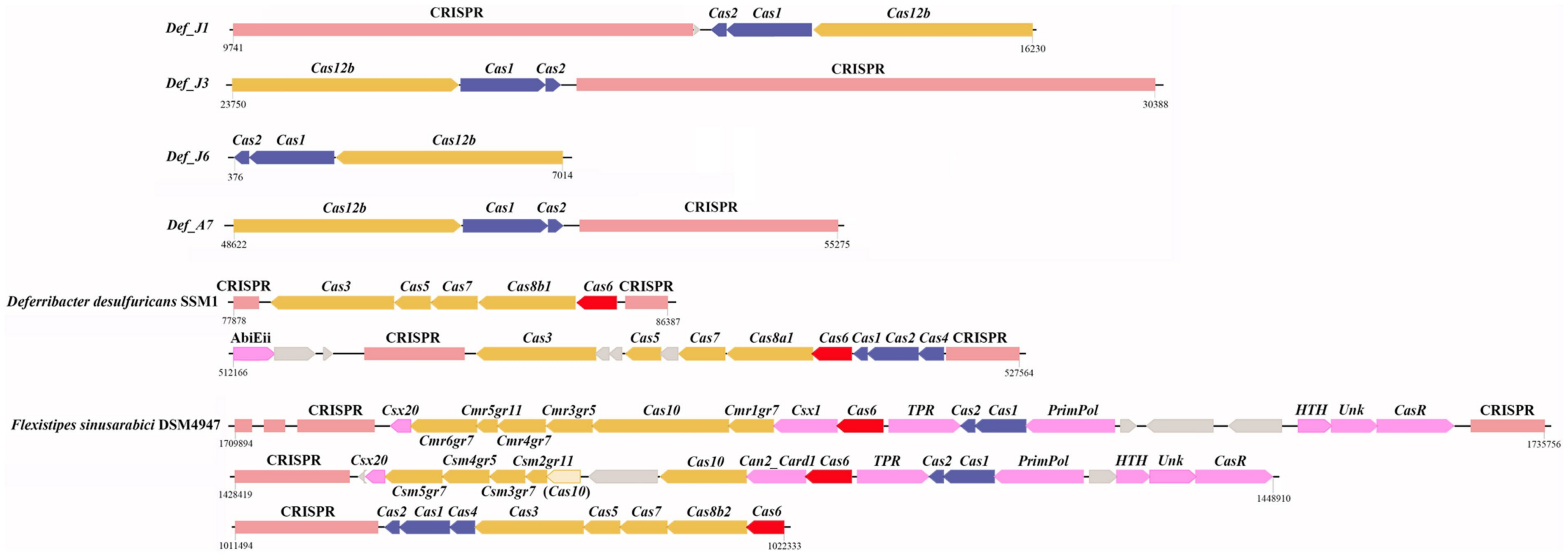
Structures of CRISPR–Cas systems. Structures of CRISPR-Cas systems in Deferribacterota and reference genomes. The CRISPR sequence is in pink. The interference module is in yellow. The adaptation module is in blue. *Cas6* is in red. Accessory genes are in purple. Genes with alignment scores below the thresholds are lighter and with parentheses around names. Unknown genes are in gray. The number represents the starting and ending positions on the contig. The arrow indicates the direction of the gene. The spacer BLAST results for spacers in the database of CRISPR/Casdb database are shown in Supplementary Table S4.

The CRISPR-cas system was investigated in six Def-MAGs, and which in *Def_J1*, *Def_J3*, *Def_J6*, and *Def_A7* consisted of three cas genes, *cas1*, *cas2*, and *cas12b*, and 70–190 spacers (Figure 5). These Def-MAGs were dominated by the type V CRISPR-cas system, belonging to the class II CRISPR-cas system due to the *cas 12b* gene, the key enzyme for type V CRISPR-cas. In contrast, in the reference genomes, only *D. desulfuricans* SSM1 and *F. sinusarabici* DSM4947 have a complete CRISPR–Cas system and belong to types I and III of class I, respectively. Furthermore, by blasting the spacer sequences of the Def-MAGs in the CRISPRCasdb database, most of them are unknown spacer sequences. A total of 17 spacer sequences were matched with identities of 92–100%, and most of them were pathogenic bacteria (Supplementary Table S4). These results suggest that the CRISPR system of hydrothermal *R. kairei* intestinal Deferribacterota genomes is likely to provide immune protection to the host against invasion by other pathogenic bacteria.

## 4. Discussion

In this study, functional annotation analysis was comparably performed between the six Def-MAGs and three free-living bacteria in the phylum Deferribacterota from the environment and one Deferribacterota from the intestinal tract of mice. Based on the features of the six Def-MAGs, Deferribacterota from the intestinal tract of *R. kairei* can utilize various carbon sources, including glycogen, sucrose, salicin, arbutin, glucose, cellobiose, and maltose.

Glycogen in bacteria plays a significant role in carbon and energy storage. It presently has been found in species such as archaea, bacteria, and heterotrophic eukaryotes (Ball et al., 2015). Glycogen is a highly soluble homogeneous polysaccharide and contains hundreds of thousands of glucose units. Glycogen can accumulate in bacteria when the carbon content exceeds that of another nutrient and limits growth (Wilson et al., 2010). During disadvantageous periods, glycogen is decomposed as a carbon and energy reserve to support the long-term survival of bacteria (Wang and Wise, 2011). Glycogen plays a central role in the widespread connectivity of various cellular pathways and can be involved in bacterial energy metabolism, environmental durability, dormancy, and virulence (McMeechan et al., 2005; Jones et al., 2008; Chandra et al., 2011; Sawers, 2016; Gupta et al., 2017; Klotz and Forchhammer, 2017). The classical pathway for glycogen synthesis is the GlgC-GlgA pathway, which generates activated glucose nucleotide diphosphate from glucose 1-phosphate *via* nucleotide diphosphate glucose pyrophosphorylase (GlgC), which then polymerizes by glycogen synthase (GlgA) to produce linear glucans. Finally, a non-reducing-end oligoglucan transfer mediated by branching enzymes (GlgB or GBE) to the 6-position of residues within a chain generates side branches that convert it to glycoge (Preiss, 2006). GBE (EC 2.4.1.18) is a determinant of glycogen structure and highly conserved (Zmasek and Godzik, 2014). Bacterial GBE belongs to the GH13 family of glucose hydrolases (α-amylase family; Feng et al., 2015). In prokaryotes, glycogen has been considered to be degraded by highly conserved glycogen phosphorylases (GlgP) and debranching enzymes (GlgX; Wilson et al., 2010; Wang and Wise, 2011). GlgP can remove non-reducing terminal glycosyl residues until four glycosyl residues remain at the branching point, then GlgX acts on the short chain by truncating the α-1,6-glycosidic bond (Dauvillée et al., 2005; Alonso-Casajús et al., 2006). The glucose 1-phosphate generated by glycogen degradation readily enters the primary generation. We found GlgP in six of the Def-MAGs, and GBE was in *Def_J1*, *Def_J3*, *Def_J6*, and *Def_A4*, indicating that Def-MAGs can participate in glycogen degradation and synthesis.

Based on the carbohydrate enzyme annotation, we found that there were nine specific enzymes in the six Def-MAGs of *R. kaire*i, namely, CBM67, CE9, GH1, GH133, GH19, GH3, GH4, GH42, and GH78, compared with the four reference genomes of Deferribacterota. Of these, CBM67 is mainly able to bind L-rhamnose. CE9 esterases catalyse the deacetylation of N-acetylglucosamine-6-phosphate to glucosamine-6-phosphate. This reaction has been demonstrated to be important for bacterial amino sugar metabolism and peptidoglycan cell wall recycling (Park, 2001; Ahangar et al., 2018). GH1 mainly includes β-glucosidases and β-galactosidases but also includes 6-phosphate-β-glucosidase and 6-phosphate-β-galactosidases, β-mannosidase, β-D-fucosidase, and β-glucuronidase (Michalska et al., 2013). Def-MAGs mainly have β-glucosidases and β-galactosidases. GH133 is mainly amylo-α 1,6-glucosidase (Stam et al., 2006). GH19 is an endo-acting enzyme that hydrolyses glycosidic bonds within chitin, a partially deacetylated chitin, with high degrees of acetylation, even though it lacks a CBM (Kawase et al., 2006). GH3 is widely distributed in bacteria, fungi, and plants and has a variety of functions, including cellulose biomass degradation, plant and bacterial cell wall remodelling, energy metabolism, and pathogen defence. It has been reported that GH3 can hydrolyse cellulose disaccharides and hydrolyse the nonreducing end β-1,4 bond of cellulose dextrins *via* β-glucosidase (Karkehabadi et al., 2018). GH4 differs from the other glycoside hydrolases in the family with different substrate specificities from each other. This family contains α-glucosidases, α-galactosidases, α-glucosidase, 6-phospho-α-glucosidases and 6-phospho-β-glucosidases. Similar to GH1, some enzymes prefer phosphorylated substrates to nonphosphorylated substrates (Henrissat and Bairoch, 1996; Henrissat and Davies, 1997). GH42 is active against lactose (Yuan et al., 2008). The only activity identified for GH78 was the hydrolysis of α-L-rhamnosides (Mutter et al., 1994). These results indicate that the intestinal Def-MAGs can hydrolyse a variety of polysaccharides and can hydrolyse and utilize residues such as cellulose and chitin that are ingested by the hydrothermal shrimp *R. kairei* as an energy source.

In this study, we found that the six Def-MAGs have a type II secretory system for transferring endotoxin and exotoxin to the extracellular system. Many gram-negative bacteria secrete toxic factors and effectors for molecular communication with their hosts through the bacterial secretory system. There are six types of protein-secreting systems in gram-negative bacteri (Costa et al., 2015). Among them, the type II secretion system is mainly centred on the general secretory pathway (GSP) gene cluster. The other reference genomes mainly contain the bacterial type VI secretory system (T6SS), which is widely found in gram-negative bacteria and is an important “weapon” of bacterial competition. Its structure is similar to that of a phage’s caudal tube, which is inverted on the inside of the bacterial cell membrane (Cianfanelli et al., 2016). T6SS is commonly produced in multiple processes associated with bacterial virulence and had shown to attack bacterial competitors or defeat host defense mechanisms to survive in competition or colonized host ecological niches (Russell et al., 2011; Basler et al., 2013). The presence of a type VI secretion system and a putative effector protein in the mouse gut of *M. schaedleri* has been reported to alter gene expression in mucosal tissue, suggesting a close interaction with the host and a possible role in inflammation (Loy et al., 2017). The type II secretory system is widely found in animal and plant pathogens and can secrete various proteins for exocytosis, which is very common in gram-negative bacteria. We also identified a type V CRISPR–Cas immune protection system in the intestinal Def-MAGs of the hydrothermal shrimp *R. kairei*, which is capable of providing immune protection to the host against multiple pathogen invasions. In conclusion, intestinal Def-MAGs of the hydrothermal shrimp *R. kairei* may enhance its viability and competitiveness in the host intestinal environment through interaction with the host and provide immune protection to the host.

Only serine, glycine, and alanine synthesis pathways are present in Def-MAGs, but other amino acid synthesis pathways are missing, suggesting that these bacteria may acquire some amino acids from the host. However, synthetic pathways of riboflavin, biotin, FMN, and FAD are found in Def-MAGs, and these cofactors can provide nutritional help for the host under extreme environments. Although it has been reported in terrestrial animals that the Deferribacterota bacterium may be a pathogen causing some diseases, we found that the intestinal Deferribacterota of the hydrothermal shrimp *R. kairei* may provide a variety of nutritional and immune protection to the host. Perhaps due to long-term coevolution, the intestinal Deferribacterota bacterium of the hydrothermal shrimp *R. kairei* has formed a mutualistic relationship with the host.

## 5. Conclusion and outlook

There is an interdependent relationship between the Deferribacterota bacterium and its host *R. kairei* in terms of material and energy, suggesting that Deferribacterota is a symbiont in the gut of *R. kairei*. The predominant occupation of Deferribacterota in the intestine of *R. kairei* plays an important role in survival. The study of the functions of Deferribacterota not only further explains the survival strategy and mechanism of blind shrimp in extreme environments but also deepens the understanding of the viability and living conditions of microorganisms.

## Data availability statement

The datasets presented in this study can be found in online repositories. The names of the repository/repositories and accession number(s) can be found in the article/Supplementary material.

## Author contributions

QL and L-SH conceived and designed the experiments. QL performed the experiments. QL, MS, F-CZ, and C-AL analysed the data. QL and L-SH wrote the manuscript with input from all other authors. L-SH directed and supervised all of the research. All authors contributed to the article and approved the submitted version.

## Funding

This study was supported by the general projects of National Natural Science Foundation of China (42176125); the Scientific research and manufacture projects of Sanya City, Grant (NO. 2020KS01); Hainan Provincial Natural Science Foundation of China, grant (322CXTD531); the major scientific and technological projects of Hainan Province (ZDKJ2019011); and the major scientific and technological projects of Hainan Province (ZDKJ2021028).

## Conflict of interest

The authors declare that the research was conducted in the absence of any commercial or financial relationships that could be construed as a potential conflict of interest.

## Publisher’s note

All claims expressed in this article are solely those of the authors and do not necessarily represent those of their affiliated organizations, or those of the publisher, the editors and the reviewers. Any product that may be evaluated in this article, or claim that may be made by its manufacturer, is not guaranteed or endorsed by the publisher.
