# Supplementary material for: Genomic evidence for the first symbiotic Deferribacterota, a novel gut symbiont from the deep-sea hydrothermal vent shrimp Rimicaris kairei
Source: Front Microbiol. 2023 Jun 29;14:1179935. doi: 10.3389/fmicb.2023.1179935 (PMC10344455; doi:10.3389/fmicb.2023.1179935)
Supplement: Supplementary file 1 [file Table_1.docx]

| **Genome features** | **Genome size（bp）** | **CheckM Completeness（%）** | **CheckM contamination（%）** | **G+C content（%）** | **Contig number** | **N50 (scaffolds)** | **CDS number** | **tRNA genes** | **rRNA genes** | **RED value** |
| --- | --- | --- | --- | --- | --- | --- | --- | --- | --- | --- |
| ***Def_J1*** | 1,364,439 | 78.19 | 0 | 49.6 | 6 | 23,370 | 1119 | 29 | 0 | 0.75 |
| ***Bac_J2*** | 2,326,929 | 100 | 0.537 | 31.3 | 13 | 55,216 | 1885 | 48 | 0 | 0.8 |
| ***Def_J3*** | 1,821,084 | 88.76 | 0.143 | 50.2 | 8 | 36,026 | 1382 | 39 | 3 | 0.47 |
| ***Fir_J4*** | 1,434,704 | 76.09 | 0.671 | 28.5 | 0 | 6,414 | 1351 | 22 | 4 | 0.76 |
| ***Def_J5*** | 927,196 | 55.33 | 0 | 49 | 0 | 4,963 | 824 | 11 | 0 | 0.46 |
| ***Def_J6*** | 2,565,562 | 90.05 | 0 | 46.4 | 10 | 20,240 | 1743 | 31 | 6 | 0.47 |
| ***Fir_J7*** | 1,602,459 | 88.12 | 0.671 | 27.8 | 7 | 281,342 | 1490 | 36 | 2 | 0.76 |
| ***Fir_J8*** | 835,327 | 87.61 | 1.127 | 26.2 | 2 | 8,872 | 713 | 27 | 0 | 0.91 |
| ***Bac_J9*** | 2,357,918 | 100 | 0.806 | 31.4 | 15 | 54,338 | 1898 | 48 | 2 | 0.8 |
| ***Spi_J10*** | 1,221,397 | 91.01 | 0.102 | 32.6 | 1 | 9,437 | 1110 | 24 | 2 | 0.69 |
| ***Cam_A1*** | 1,339,737 | 69.2 | 1.27 | 41.7 | 1 | 4,199 | 1358 | 10 | 0 | 0.96 |
| ***Cam_A2*** | 2,015,390 | 95.52 | 0.406 | 26.3 | 2 | 15,218 | 2016 | 29 | 0 | 0.82 |
| ***Cam_A3*** | 1,073,521 | 56.14 | 1.653 | 38.7 | 0 | 5,107 | 1095 | 15 | 0 | 0.98 |
| ***Def_A4*** | 1,567,595 | 75.13 | 0 | 49.3 | 2 | 10,807 | 1309 | 32 | 18 | 0.75 |
| ***Cam_A5*** | 1,664,110 | 89.75 | 2.937 | 38.3 | 0 | 10,177 | 1681 | 27 | 0 | 0.85 |
| ***Bac_A6*** | 2,529,059 | 99.46 | 1.344 | 31.2 | 15 | 49,623 | 2071 | 48 | 0 | 0.8 |
| ***Def_A7*** | 1,248,453 | 71.92 | 1.754 | 50.7 | 7 | 38,451 | 935 | 17 | 1 | 0.46 |
| ***Cam_A8*** | 1,253,562 | 57.05 | 2.586 | 41.9 | 0 | 4,973 | 1249 | 10 | 0 | 0.96 |

**Table S1. Intestinal metagenomic information of the hydrothermal blind shrimp *R. kairei*.**
