# Supplementary material for: Genomic evidence for the first symbiotic Deferribacterota, a novel gut symbiont from the deep-sea hydrothermal vent shrimp Rimicaris kairei
Source: Front Microbiol. 2023 Jun 29;14:1179935. doi: 10.3389/fmicb.2023.1179935 (PMC10344455; doi:10.3389/fmicb.2023.1179935)
Supplement: Supplementary file 2 [file Table_2.docx]

**Table S2. Integrity statistics of each metabolic pathway in KEGG heat map.**

|  | **Metabolic Pathway** | ***Def_J1*** | ***Def_J3*** | ***Def_J5*** | ***Def_J6*** | ***Def_A4*** | ***Def_A7*** | ***Mucispirillum schaedleri* ASF457** | ***Deferribacter autotrophicus*** | ***Deferribacter desulfuricans* SSM1** | ***Flexistipes sinusarabici* DSM4947** |
| --- | --- | --- | --- | --- | --- | --- | --- | --- | --- | --- | --- |
| Carbon metabolism | glycolysis | 0.67 | 0.78 | 0.22 | 0.78 | 0.56 | 0.78 | 0.78 | 0.78 | 0.78 | 0.78 |
|  | gluconeogenesis | 0.67 | 0.78 | 0.33 | 0.78 | 0.56 | 0.78 | 0 | 0.78 | 0.78 | 0.78 |
|  | TCA Cycle | 0.12 | 0.12 | 0 | 0.12 | 0 | 0.12 | 0.38 | 0.62 | 0.62 | 0.62 |
| oxidative phosphorylaion | NAD(P)H-quinone oxidoreductase | 0 | 0 | 0 | 0 | 0 | 0 | 0 | 0.07 | 0.07 | 0.07 |
|  | NADH-quinone oxidoreductase | 0.35 | 0.42 | 0 | 0.42 | 0.35 | 0 | 0 | 0.84 | 0.84 | 0.84 |
|  | F-type ATPase | 1 | 1 | 0.625 | 1 | 1 | 1 | 0.875 | 1 | 1 | 1 |
|  | V-type ATPase | 0 | 0 | 0 | 0 | 0 | 0 | 0 | 0 | 0 | 0.11 |
|  | Cytochrome c oxidase, cbb3-type | 0.25 | 0.25 | 0.25 | 0.25 | 0.25 | 0.25 | 0 | 0 | 0 | 0 |
|  | Cytochrome bd complex | 0 | 0 | 0 | 0 | 0 | 0 | 0 | 1 | 1 | 1 |
| carbon fixation | Wood-Ljungdahl | 0 | 0 | 0 | 0 | 0 | 0 | 0 | 0.17 | 0 | 0 |
|  | 3-Hydroxypropionate Bicycle | 0.06 | 0.06 | 0 | 0.06 | 0 | 0.06 | 0.06 | 0.18 | 0.18 | 0.18 |
|  | 4-Hydroxybutyrate/3-hydroxypropionate | 0 | 0 | 0 | 0 | 0 | 0 | 0.2 | 0.3 | 0.3 | 0.3 |
| carbon degradation | D-galacturonate epimerase | 0 | 0 | 0 | 0 | 0 | 0 | 0 | 1 | 1 | 1 |
|  | beta-N-acetylhexosaminidase | 1 | 1 | 0 | 1 | 0 | 1 | 0 | 0 | 0 | 1 |
|  | beta-glucosidase | 0 | 1 | 0 | 1 | 1 | 0 | 0 | 0 | 0 | 0 |
| nitrogen metabolism | hydroxylamine oxidation | 0 | 0 | 0 | 0 | 0 | 0 | 0 | 1 | 1 | 1 |
|  | nitrite oxidation | 0 | 0 | 0 | 0 | 0 | 0 | 0 | 0 | 1 | 0 |
|  | dissim nitrate reduction | 0 | 0 | 0 | 0 | 0 | 0 | 0 | 0 | 1 | 0 |
|  | DNRA | 1 | 0 | 1 | 0 | 1 | 0 | 0 | 0 | 0 | 0 |
| sulfur metabolism | thiosulfate/polysulfide reductase | 0 | 0 | 0 | 0 | 0 | 0 | 0 | 0.33 | 0.33 | 0.33 |
|  | sulfide oxidation | 1 | 1 | 0 | 1 | 1 | 1 | 1 | 0 | 0 | 0 |
|  | DMSO reductase | 0 | 0 | 0 | 0 | 0 | 0 | 0.33 | 0 | 0 | 0 |
| Hydrogen redox | NiFe hydrogenase Hyd-1 | 0 | 0 | 0 | 0 | 0 | 0 | 0.66 | 0.99 | 0.99 | 0.99 |
| vitamin biosynthesis | thiamin biosynthesis | 0.27 | 0.27 | 0.18 | 0.27 | 0.27 | 0.27 | 0.73 | 0.73 | 0.82 | 0.82 |
|  | riboflavin biosynthesis | 1 | 1 | 0.75 | 1 | 1 | 1 | 1 | 1 | 1 | 1 |
|  | cobalamin biosynthesis | 0 | 0 | 0 | 0 | 0 | 0 | 0.75 | 0.75 | 0.75 | 0.12 |
| transporters | transporter: phosphonate | 0 | 0 | 0 | 0 | 0 | 0 | 0 | 0.33 | 0.33 | 0.33 |
|  | transporter: phosphate | 1 | 1 | 0.25 | 1 | 0.25 | 1 | 0 | 1 | 1 | 1 |
| cell motility | Flagellum | 0.83 | 0.87 | 0.39 | 0.87 | 0.83 | 0.78 | 0.91 | 1 | 1 | 0 |
|  | Chemotaxis | 0.25 | 0.38 | 0.12 | 0.5 | 0.38 | 0.5 | 0.88 | 0.88 | 0.88 | 0.12 |
| Methane metabolism | Methanogenesis via CO_2_ | 0 | 0 | 0 | 0 | 0 | 0 | 0.05 | 0.05 | 0.05 | 0 |
|  | Coenzyme B/Coenzyme M regeneration | 0 | 0 | 0 | 0 | 0 | 0 | 0 | 0.2 | 0.2 | 0.2 |
| Photosynthesis | Retinal biosynthesis | 0 | 0 | 0 | 0 | 0 | 0 | 0.25 | 0.25 | 0.25 | 0 |
| mixed acid fermentation | Mixed acid: Formate to CO_2_ & H_2_ | 0 | 0 | 0 | 0 | 0 | 0 | 0.334 | 0.501 | 0.501 | 0.501 |
|  | Mixed acid: Acetate | 0 | 0 | 0 | 0 | 0 | 0 | 1 | 1 | 0.5 | 1 |
|  | Mixed acid: PEP to Succinate via OAA, malate & fumarate | 0.458 | 0.458 | 0 | 0.458 | 0.375 | 0.458 | 0.3955 | 0.458 | 0.458 | 0.3955 |
| Competence-related DNA transporter | Competence-related core components | 0.07 | 0.07 | 0.07 | 0.07 | 0.07 | 0 | 0.07 | 0.07 | 0.07 | 0.07 |
| Anaplerotic Reactions | Anaplerotic genes | 0.25 | 0.5 | 0 | 0.5 | 0.25 | 0.5 | 0.5 | 0.75 | 0.75 | 0.75 |
| Bacterial secretion systems | Type I Secretion | 0 | 0 | 0 | 0 | 0 | 0 | 0 | 0.33 | 0.33 | 0.33 |
|  | Type II Secretion | 0.3076 | 0.3845 | 0.1538 | 0.3076 | 0.3845 | 0.3845 | 0.3076 | 0.0769 | 0.3845 | 0.3845 |
|  | Type IV Secretion | 0 | 0 | 0 | 0 | 0 | 0 | 0.083 | 0.083 | 0 | 0 |
|  | Type VI Secretion | 0 | 0 | 0 | 0 | 0 | 0 | 0.555 | 0.111 | 0.111 | 0 |
|  | Sec-SRP | 0.83 | 0.83 | 0 | 0.747 | 0.83 | 0 | 0.913 | 0.83 | 0.83 | 0.83 |
|  | Twin Arginine Targeting | 0 | 0 | 0 | 0 | 0 | 0 | 0.5 | 0.75 | 0.75 | 0.75 |
| amino acid metabolism | Serine pathway/formaldehyde assimilation | 0.2 | 0.3 | 0.2 | 0.3 | 0.2 | 0.2 | 0.3 | 0.4 | 0.4 | 0.4 |
| metal transporters | Cobalt transporter CbiMQ | 0 | 0 | 0 | 0 | 0 | 0 | 1 | 0.5 | 0.5 | 0.5 |
|  | Copper transporter CopA | 0 | 1 | 0 | 1 | 0 | 1 | 1 | 1 | 1 | 1 |
|  | Ferric iron ABC-type substrate-binding AfuA | 0 | 0 | 0 | 0 | 0 | 0 | 0 | 0 | 0 | 1 |
| amino acid biosynthesis | histidine | 0 | 0 | 0 | 0 | 0 | 0 | 1 | 1 | 1 | 1 |
|  | arginine | 0 | 0 | 0 | 0 | 0 | 0 | 1 | 1 | 1 | 1 |
|  | lysine | 0 | 0 | 0 | 0 | 0 | 0 | 1 | 1 | 1 | 1 |
|  | serine | 1 | 1 | 1 | 1 | 1 | 0 | 1 | 1 | 1 | 1 |
|  | threonine | 0 | 0 | 0 | 0 | 0 | 0 | 1 | 1 | 1 | 1 |
|  | glutamine | 0 | 0 | 0 | 0 | 0 | 0 | 1 | 1 | 1 | 1 |
|  | cysteine | 0 | 0 | 0 | 0 | 0 | 0 | 1 | 1 | 1 | 1 |
|  | glycine | 1 | 1 | 1 | 1 | 1 | 0 | 1 | 1 | 1 | 1 |
|  | proline | 0 | 0 | 0 | 0 | 0 | 0 | 1 | 1 | 1 | 1 |
|  | alanine | 1 | 1 | 0 | 1 | 1 | 1 | 0 | 1 | 1 | 1 |
|  | valine | 0 | 0 | 0 | 0 | 0 | 0 | 0.83 | 0.83 | 0.83 | 0.83 |
|  | methionine | 0 | 0 | 0 | 0 | 0 | 0 | 1 | 1 | 1 | 1 |
|  | phenylalanine | 0 | 0 | 0 | 0 | 0 | 0 | 1 | 1 | 1 | 1 |
|  | isoleucine | 0 | 0 | 0 | 0 | 0 | 0 | 0.83 | 0.83 | 0.83 | 0.83 |
|  | leucine | 0 | 0 | 0 | 0 | 0 | 0 | 1 | 1 | 1 | 1 |
|  | tryptophan | 0 | 0 | 0 | 0 | 0 | 0 | 0 | 1 | 1 | 1 |
| Starch and sucrose metabolism | starch/glycogen synthesis | 0.66 | 0.66 | 0.33 | 0.66 | 0.66 | 0.33 | 0.66 | 0 | 0 | 0 |
|  | starch/glycogen degradation | 1 | 1 | 1 | 1 | 1 | 1 | 1 | 0 | 0 | 0 |
| Butanoate fermentation | polyhydroxybutyrate synthesis | 0 | 0 | 0 | 0 | 0 | 0 | 0.167 | 0.167 | 0.167 | 0.167 |
| Two component system | bidirectional polyphosphate | 0.5 | 0.5 | 0.5 | 0.5 | 0.5 | 0.5 | 1 | 0.5 | 0.5 | 0.5 |
| carotenoid biosynthesis | end-product myxoxanthophylls | 0 | 0 | 0 | 0 | 0 | 0 | 0 | 0.33 | 0 | 0 |
|  | MEP-DOXP pathway | 0.71 | 0.71 | 0.426 | 0.71 | 0.71 | 0.71 | 0.71 | 0.71 | 0.71 | 0.71 |
