# Supplementary material for: Genomic evidence for the first symbiotic Deferribacterota, a novel gut symbiont from the deep-sea hydrothermal vent shrimp Rimicaris kairei
Source: Front Microbiol. 2023 Jun 29;14:1179935. doi: 10.3389/fmicb.2023.1179935 (PMC10344455; doi:10.3389/fmicb.2023.1179935)
Supplement: Supplementary file 3 [file Table_3.docx]

**Table S3. Statistics of carbohydrate active enzyme genes in *R. kairei* gut Deferribacterota MAGs and reference genomes.**

| **Type** | **CAZymes** | ***Def_J1*** | ***Def_J3*** | ***Def_J5*** | ***Def_J6*** | ***Def_A4*** | ***Def_A7*** | ***Mucispirillum schaedleri*** | ***Deferribacter desulfuricans*** | ***Deferribacter autotrophicus*** | ***Flexistipes sinusarabici*** |
| --- | --- | --- | --- | --- | --- | --- | --- | --- | --- | --- | --- |
| **Auxiliary Activities (AAs)** | **AA4** | 0 | 0 | 0 | 0 | 0 | 0 | 0 | 1 | 0 | 0 |
| **Carbohydrate-Binding Modules (CBMs)** | **CBM67** | 0 | 2 | 0 | 1 | 1 | 0 | 0 | 0 | 0 | 0 |
|  | **CBM13** | 0 | 0 | 0 | 0 | 0 | 0 | 1 | 0 | 0 | 0 |
| **Carbohydrate Esterases (CEs)** | **CE11** | 1 | 1 | 1 | 1 | 1 | 1 | 1 | 1 | 1 | 1 |
|  | **CE9** | 1 | 1 | 1 | 1 | 1 | 1 | 0 | 0 | 0 | 0 |
|  | **CE4** | 0 | 0 | 0 | 0 | 0 | 0 | 0 | 1 | 1 | 1 |
| **Glycosyl Transferases (GTs)** | **GT9** | 4 | 4 | 1 | 4 | 3 | 3 | 6 | 3 | 3 | 3 |
|  | **GT5** | 1 | 1 | 1 | 1 | 1 | 1 | 2 | 0 | 0 | 0 |
|  | **GT35** | 1 | 1 | 0 | 1 | 1 | 1 | 1 | 0 | 0 | 0 |
|  | **GT66** | 1 | 1 | 1 | 1 | 1 | 0 | 1 | 1 | 1 | 1 |
|  | **GT51** | 1 | 1 | 1 | 1 | 2 | 1 | 2 | 1 | 1 | 1 |
|  | **GT28** | 1 | 1 | 1 | 1 | 1 | 1 | 1 | 2 | 1 | 1 |
|  | **GT30** | 1 | 1 | 1 | 1 | 1 | 1 | 1 | 1 | 1 | 1 |
|  | **GT19** | 1 | 1 | 1 | 1 | 1 | 0 | 1 | 1 | 1 | 1 |
|  | **GT2** | 0 | 7 | 0 | 4 | 2 | 0 | 11 | 5 | 4 | 4 |
|  | **GT4** | 0 | 0 | 0 | 0 | 0 | 0 | 1 | 9 | 9 | 9 |
|  | **GT83** | 0 | 0 | 0 | 0 | 0 | 0 | 1 | 2 | 2 | 2 |
|  | **GT81** | 0 | 0 | 0 | 0 | 0 | 0 | 1 | 1 | 1 | 1 |
| **Glycoside Hydrolases (GHs)** | **GH13** | 2 | 2 | 0 | 2 | 1 | 1 | 0 | 1 | 1 | 1 |
|  | **GH23** | 1 | 1 | 0 | 1 | 1 | 1 | 2 | 3 | 4 | 4 |
|  | **GH3** | 1 | 1 | 0 | 1 | 0 | 1 | 0 | 0 | 0 | 0 |
|  | **GH19** | 1 | 1 | 0 | 1 | 0 | 1 | 0 | 0 | 0 | 0 |
|  | **GH133** | 1 | 1 | 0 | 1 | 1 | 1 | 0 | 0 | 0 | 0 |
|  | **GH4** | 0 | 2 | 0 | 1 | 0 | 0 | 0 | 0 | 0 | 0 |
|  | **GH1** | 0 | 1 | 0 | 1 | 1 | 0 | 0 | 0 | 0 | 0 |
|  | **GH42** | 0 | 0 | 1 | 0 | 1 | 0 | 0 | 0 | 0 | 0 |
|  | **GH78** | 0 | 2 | 0 | 1 | 1 | 0 | 0 | 0 | 0 | 0 |
|  | **GH103** | 0 | 0 | 0 | 0 | 0 | 0 | 1 | 1 | 1 | 1 |
|  | **GH57** | 0 | 0 | 0 | 0 | 0 | 0 | 3 | 0 | 0 | 0 |
|  | **GH153** | 0 | 0 | 0 | 0 | 0 | 0 | 1 | 1 | 1 | 1 |
|  | **GH109** | 0 | 0 | 0 | 0 | 0 | 0 | 0 | 1 | 1 | 1 |
|  | **GH130** | 0 | 0 | 0 | 0 | 0 | 0 | 0 | 1 | 1 | 1 |
|  | **GH114** | 0 | 0 | 0 | 0 | 0 | 0 | 0 | 0 | 1 | 1 |
