# Supplementary material for: Genomic evidence for the first symbiotic Deferribacterota, a novel gut symbiont from the deep-sea hydrothermal vent shrimp Rimicaris kairei
Source: Front Microbiol. 2023 Jun 29;14:1179935. doi: 10.3389/fmicb.2023.1179935 (PMC10344455; doi:10.3389/fmicb.2023.1179935)
Supplement: Supplementary file 4 [file Table_4.docx]

**Table S4. Spacers blast in CRISPRCasdb database results.**

| Bacteria | Spacers | Nucleotides match | Bit score | identity % | E-value |
| --- | --- | --- | --- | --- | --- |
| *Def_J1* |  |  |  |  |  |
| Candidatus Kapabacteria bacterium | TCAAACCAACAAAATCAAACGTTAACACTACAAC | 23/34 | 34.2 | 92% | 0.008 |
| Ignavibacteria bacterium | TTACTAATCAAAAAAAAGGAAGGTAAAAATGGAAGGTAAA | 17/40 | 34.2 | 100% | 0.009 |
| Aggregatibacter actinomycetemcomitans 624 | AGTATCCTAAATCATTGATATTTTTCCTA | 17/32 | 34.2 | 100% | 0.008 |
| *Def_J3* |  |  |  |  |  |
| Nakamurella multipartita DSM 44233 | ACCCTCCAGGAGCGGGGGCGTGCGTGGCGC | 17/32 | 34.2 | 100% | 0.009 |
| Pasteurella muftocida FDAARGOS_218  Pasteurella muitocida subsp.gallicida P1059 | TTGAACAAATGACATTGGATGAATTATCAACTT  AAGTTGATAATTCATCCAATTCATTTGTTCAA | 17/33 | 34.2 | 100% | 0.008 |
| Burkholderiaceae bacterium | CCATAGGAGGCGTCGCGACGCGTGGTTCGTAAGG | 17/36 | 34.2 | 100% | 0.008 |
| Salmonella enterica subsp.salamae LHICA_SA2 | GATGCGATGGGGAATATTGTTAAAGTGGTTTAC | 17/33 | 34.2 | 100% | 0.008 |
| Acinetobacter baumannii J9 | GCAACGIGTACGICTAGCTATATATGCTGAACCATCA | 17/37 | 34.2 | 100% | 0.008 |
| Acinetobacter baumanni A1296  Acinetobedier beumanni B2 | GTGTACGTCTAGCTATATATGCTGAACCATCA  TGATGGTTCAGCATATATAGCTAGACGTACAC | 17/32 | 34.2 | 100% | 0.008 |
| Pseudomonas aeruginosa PES_P749 | ACGGGCGGGCCGTCGGTGCGATGCTGACCGT | 17/32 | 34.2 | 100% | 0.009 |
| Arachidicoccus sp. KIS59-12 | GACTCAAAGAATTTAATGTAATCCTGAATAATTGC | 17/35 | 34.2 | 100% | 0.010 |
| Runella sp. SP2 | AAAATGTAATCTAAATTCCATAATTCAGGATTACATTTT | 17/39 | 34.2 | 100% | 0.010 |
| Halomonas sulfidaeris ATCC BAA-803 | GATGCGCTTACGCTTGGGGTTGTCCAGCACGTAGCGTC | 21/38 | 36.2 | 95% | 0.002 |
| *Def_J6* |  |  |  |  |  |
| Candidatus Thiodictyon syntrophicum Cad16T | ACGAAGTCGGTGGCGTCGGCGATGGTGATGGTGAGCC | 21/37 | 36.2 | 95% | 0.003 |
| *Def_A7* |  |  |  |  |  |
| Koleobacter methoxystrophiousNRmbB1 | TCGCATCGAGCTTCTTCCTTTTCGCTAGCAAGTCAA | 17/36 | 34.2 | 100% | 0.009 |
