# Supplementary figures and images for: Genomic evidence for the first symbiotic Deferribacterota, a novel gut symbiont from the deep-sea hydrothermal vent shrimp Rimicaris kairei
Source: Front Microbiol. 2023 Jun 29;14:1179935. doi: 10.3389/fmicb.2023.1179935 (PMC10344455; doi:10.3389/fmicb.2023.1179935)

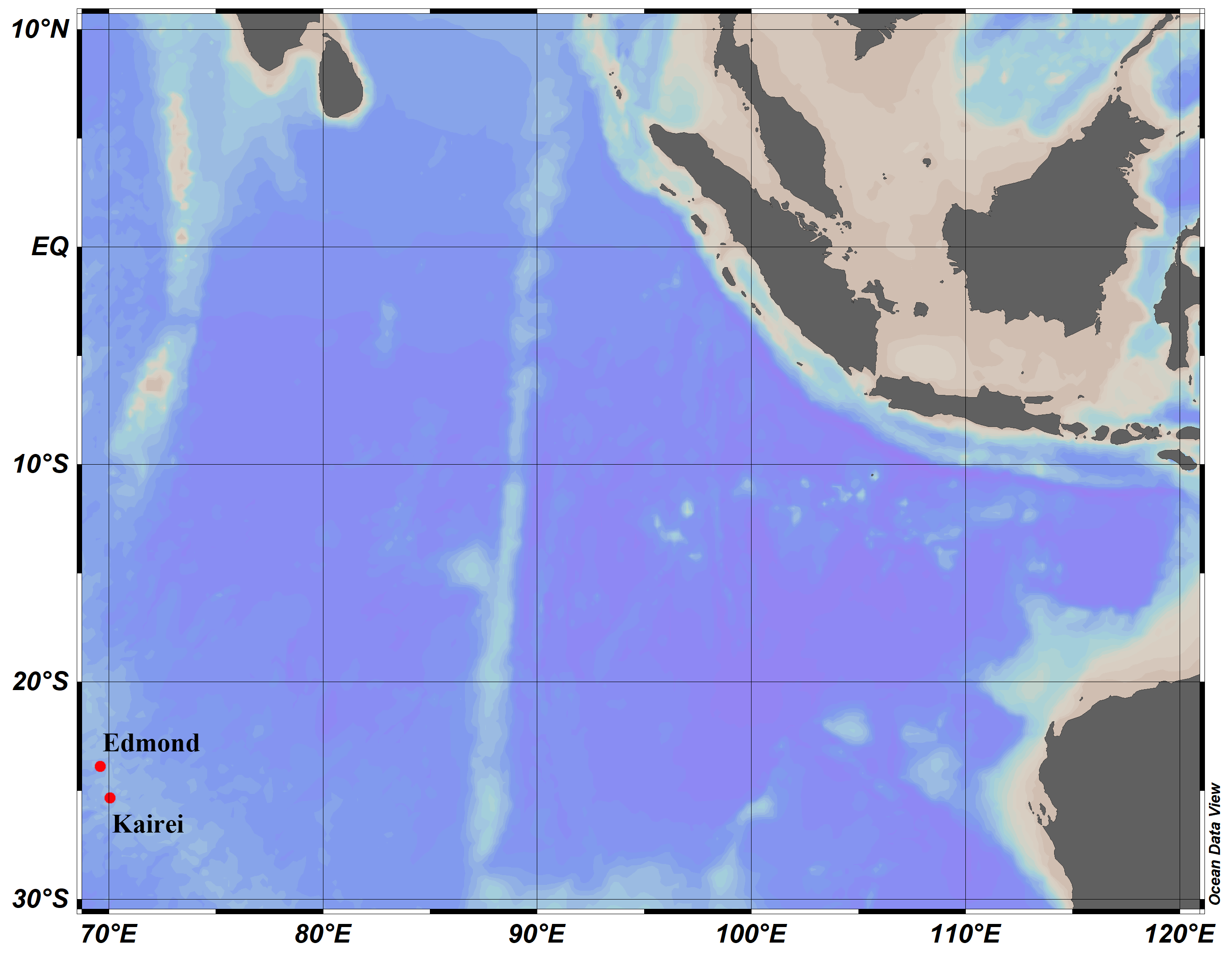

Supplement: Supplementary file 5 [file Image_1.TIF]

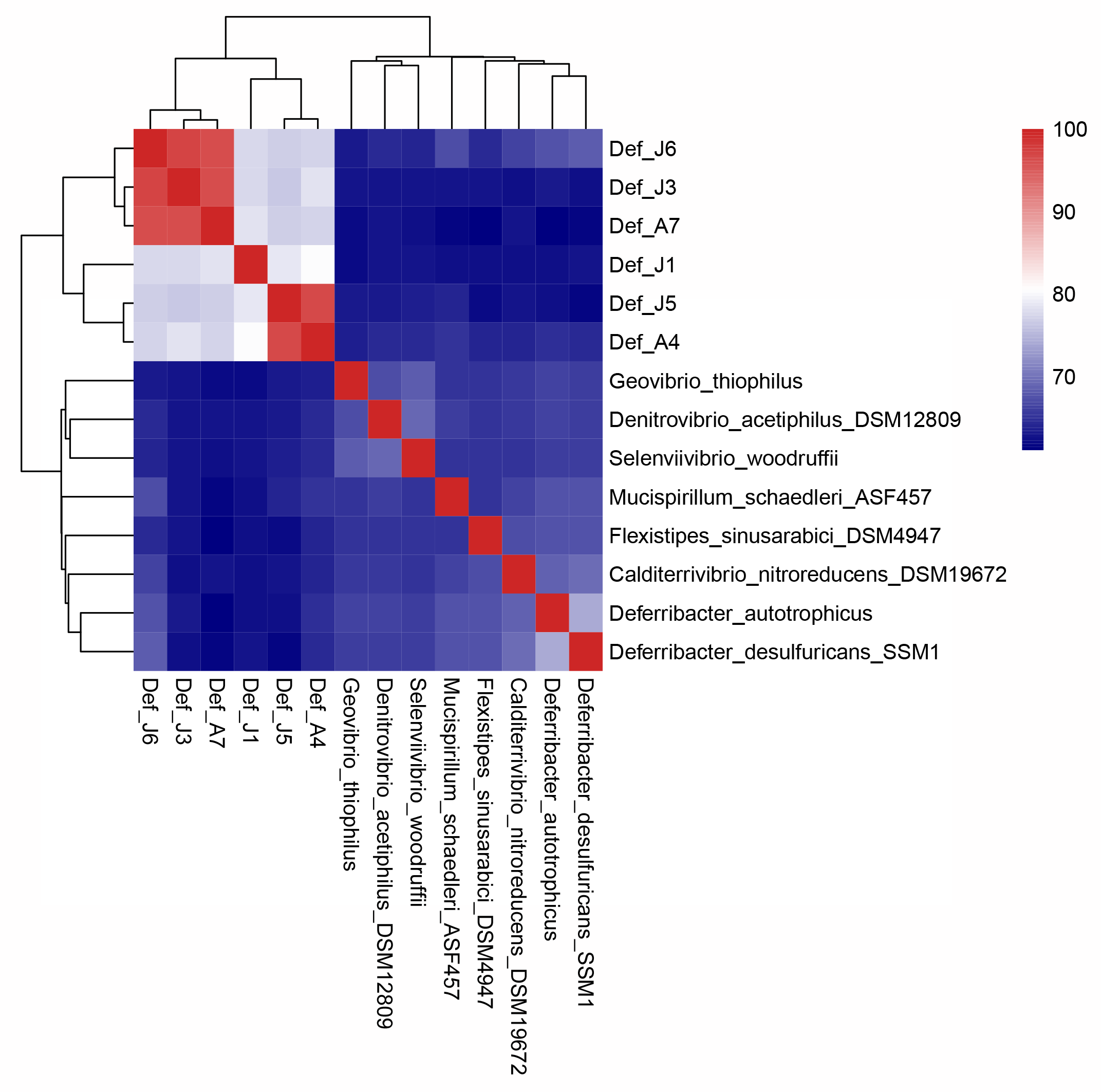

Supplement: Supplementary file 6 [file Image_2.TIF]
